# The Prospect and Challenges to the Flow of Liquid Biopsy in Africa

**DOI:** 10.3390/cells8080862

**Published:** 2019-08-09

**Authors:** Dada Oluwaseyi Temilola, Martha Wium, Tangbadioa Herve Coulidiati, Henry Ademola Adeola, Giuseppina Maria Carbone, Carlo Vittorio Catapano, Luiz Fernando Zerbini

**Affiliations:** 1 International Centre for Genetic Engineering and Biotechnology (ICGEB), Cape Town 7925, South Africa; 2 Integrative Biomedical Sciences Division, Faculty of Health Sciences, University of Cape Town, Cape Town 7925, South Africa; 3 Training and Research unit in Sciences and Technology, University Norbert Zongo, P.O. Box 376, Koudougou 376, Burkina Faso; 4 Division of Dermatology, Department of Medicine, Faculty of Health Sciences and Groote Schuur Hospital, University of Cape Town, Cape Town 7925, South Africa; 5 Institute of Oncology Research, Università della Svizzera Italiana, Via Vincenzo Vela 6, CH-6500 Bellinzona, Switzerland

**Keywords:** Africa, cancer, cell-free DNA, circulating tumor cell, circulating RNA, liquid biopsy, non-invasive

## Abstract

Liquid biopsy technologies have the potential to transform cancer patient management as it offers non-invasive diagnosis and real-time monitoring of disease progression and treatment responses. The use of liquid biopsy for non-invasive cancer diagnosis can have pivotal importance for the African continent where access to medical infrastructures is limited, as it eliminates the need for surgical biopsies. To apply liquid biopsy technologies in the African setting, the influence of environmental and population genetic factors must be known. In this review, we discuss the use of circulating tumor cells, cell-free nucleic acids, extracellular vesicles, protein, and other biomolecules in liquid biopsy technology for cancer management with special focus on African studies. We discussed the prospect, barriers, and other aspects that pose challenges to the use of liquid biopsy in the African continent.

## 1. Introduction

Cancer is a growing public health threat globally. GLOBOCAN 2018 data showed an overall increase in cancer cases worldwide with 18.1 million new cases and 9.6 million cancer deaths in 2018 [1]. Africa and Asia were showed to have a higher proportion of cancer mortality in relation to the proportion of incident cases when compared with other regions of the world [1]. 

The incidence and mortality rate of cancer differ across regions and between sexes. Globally, lung cancer had the highest incidence among males in 2018, with prostate cancer having the highest mortality burden among African men. Breast cancer still has the highest incidence and mortality burden among women worldwide [1]. The incidence and mortality rate of breast cancer have remained relatively unchanged over the years in many developed countries. In many parts of Africa, Asia, and South America the incidence of breast cancer is, however increasing rapidly, with Africa having the highest age-standardized mortality rate globally [1,2,3,4].

The rising burden of cancer in Africa has been attributed to factors such as inadequate health care facilities, poor access to quality and affordable health care, as well as inadequate infrastructure to support African-based research [5]. Furthermore, most cancers are diagnosed late in Africa which in turn worsen the prognosis [6,7].

**Figure 1:**
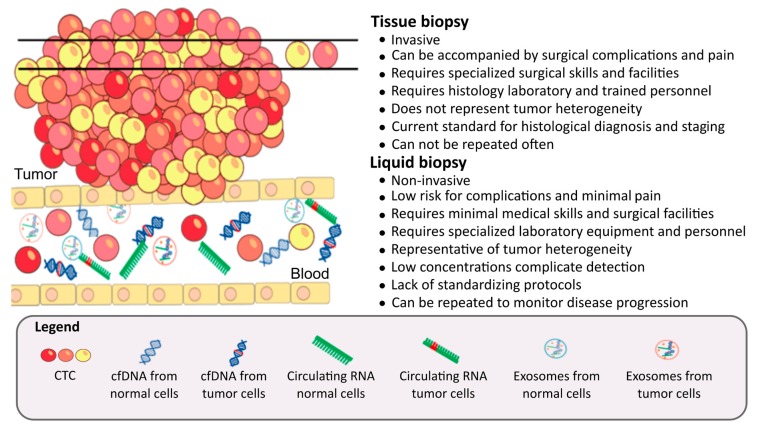
The advantages and disadvantages of a tissue biopsy in comparison with a liquid biopsy for cancer diagnosis and treatment. The illustration shows a tumor consisting of heterogeneous cells (represented by different colors). During a tissue biopsy, a small section of tissue is removed; this section may not represent the heterogeneity of the tumor. Tumor cells can undergo epithelial-to-mesenchymal transition (EMT) and enter the blood (CTC). Small molecules are also released from tumor cells into the blood, these include cfDNA, RNA, and exosomes. Tumor-specific alterations in CTCs, cfDNA, RNA, and exosomes found in blood (liquid biopsy) can be utilized to diagnose and treat cancer.

Tissue biopsy, the established method of cancer diagnosis, is invasive and can be accompanied by various surgical complications. Tissue biopsy reflects a small section of the tissue and may miss important diagnostic details. It may be inadequate for a complete genomic profile of a patient’s tumors because regions within and between primary and metastatic tumors can have different genomic mutations [8]. In a liquid biopsy, cancer is diagnosed or monitored by analyzing body fluids such as blood, urine, or saliva [9]. Liquid biopsy is based on detecting tumor cells or tumor-derived molecules (DNA, RNA, exosomes, and protein) that were released from tumors into circulation (Figure 1). Improved diagnosis, early detection, and better monitoring of disease progression and treatment response are imperative in Africa due to the overall rising burden of cancer throughout the continent. Invasive diagnostic procedures are a barrier to overcome due to surgical risk, costs, limited access, and poor compliance by the population. Therefore, development and implementation of non-invasive liquid biopsy methodologies for cancer management are a top priority for the next decades for basic and clinical scientists in Africa. In addition to being used in cancer management, liquid biopsy tests are also clinically used to detect fetal chromosomal abnormalities during pregnancies and monitor organ transplants [10]. 

There is presently an increasing number of studies on circulating tumor molecules in diagnosis and prognosis of cancers. Studies on the role of circulating molecules in cancer diagnosis started globally in the late 1990s [11,12,13,14] but African-based studies started only in late 2000 (Table 1). The majority of African-based studies were done in Egypt, with a few other studies from Tunisia, South Africa, Gambia, Cameroon, and Senegal (Table 1). Importantly, the causes of cancer differ in different populations. Distinct pathogens, carcinogens, dietary habits, social conditions, and genetic background may influence tumorigenesis depending on population and geographical settings. The genetic and epigenetic variation from population to population may lead to ample variations in natural history and clinical outcome across different populations. For example, some cancers, such as prostate cancers, are more aggressive in the African population [15]. Also, more cancers in Africa and Asia are related to infective pathogens than in other continents. This requires that more African-based studies are done to validate the applicability of circulating biomarkers and liquid biopsy technologies in diagnosis and treatment of cancer in Africa. Host genetics, tumor genetics, and epigenetic variations need to be explored and taken into account to identify population-specific cancer biomarkers in liquid biopsy adapted and optimized for diagnostic use in African countries. The process of optimization of these cutting-edge technologies should also imperatively aim at reducing costs and increasing affordable access throughout the African continent.

There are three main types of circulating molecules investigated as tumor biomarkers through liquid biopsy procedures: Circulating tumor cells (CTC), tumor-released nucleic acids like DNA and RNA, and small extracellular vesicles or exosomes, Table 2. Here we aim at reviewing studies on the role of circulating tumor molecules in the diagnosis and treatment of cancer, with a particular focus in the African continent. This review will also discuss the prospect and challenges associated with the use of circulating tumor molecules in liquid biopsy for diagnosis and treatment of cancer in Africa.

## 2. Component of Liquid Biopsy

### 2.1. Circulating Tumor Cells

Cancer deaths are mostly due to tumor metastasis [77]. Tumor cells dislodge from the primary tumor and use the blood and lymph system as highways to travel to other parts of the body, where they invade and form metastatic tumors. Our understanding of tumor cell migration is far from complete. It is associated with epithelial-to-mesenchymal transition (EMT) and governed by numerous genes and proteins in which genetic aberrations can accelerate or decelerate cancer progression. The potential of these circulating tumor cells (CTCs) in cancer diagnosis and treatment is twofold. Firstly, it can be used as a diagnostic marker to predict disease progression and survival in metastatic cancer [78,79,80], indicating treatment failure [81,82], distinguish benign from malignant growth [83], and in early-stage cancer diagnosis [84,85]. Secondly, with improvements in sequencing technologies, it also provides a window into the genetic landscape of diseases which could be used to stratify patient treatment [86].

Several methods are available for the detection of CTCs in the blood (reviewed in [87]). The only US Food and Drug Administration (FDA) approved method to quantify CTCs is the CellSearch system (Verdex LLC, San Diego, CA, USA). This method has been approved to monitor treatment effectiveness in metastatic breast, prostate, and colorectal cancer patients. The CellSearch system is sensitive, robust, and can detect a single CTCs in 7.5 mL of blood. CTCs are stable for about 96 h at room temperature when blood is collected in CellSave preservative tubes [88,89], making shipment at room temperature from remote locations viable. A limitation of this method is that the enrichment step captures cells expressing the epithelial cell adhesion molecule (EpCAM) on their surface and not all CTCs express EpCAM. Some CTCs exhibit stem cell-like or normal-like characteristics with no EpCAM surface protein while others undergo EMT transition with reduce or loss of EpCAM surface protein [90,91,92]. These CTCs will be undetectable by the CellSearch technology. Another concern is false positive results, circulating cells that are EpCAM-, CK8-, CK18-, and CK19 -positive have been detected in patients with benign inflammatory colon diseases, based on nuclear morphology these cells were consistent with benign gland [93]. It should be noted that no CTCs were detected in healthy control patients. In blood spiking experiments with carcinoma cells that have high levels of EpCAM, the sensitivity to detect CTCs is higher than 85% [94,95], but with cells that express low levels of EpCAM, the sensitivity is about 42% [96].

Using the CellSearch method, 5 or more CTCs for metastatic breast and prostate cancer and 3 or more in metastatic colorectal cancer were associated with shorter progression-free and overall survival [78,79,80]. CTC count is a more reproducible predictor of overall survival than traditional methods such as radiology [97] or prostate specific antigen (PSA) in prostate cancer [98].

Cancer treatment decisions are complicated by the disparity in responses observed between patients. Discontinuing ineffective treatment earlier may decrease morbidity due to toxicity, allow alternative treatment, and reduce treatment cost. Cancer treatment leads to a reduction in CTC count [99]. Assessing the CTC count before and after treatment can predict the outcome of treatment in castration-resistant prostate cancer [81]. CTC count is superior to traditional radiology because it can detect treatment failure earlier and more accurately [97]. A phase II trial on advanced colorectal cancer has shown that CTC count can also be used in the stratification of treatment. Patients with high CTC count may benefit most from an intensive multidrug regimen, which is usually associated with high toxicity. This can be avoided in patients with a low CTC count [100].

CTC count has also been recently used to differentiate between benign and malignant tumors. Lung lesions on a PET/CT-scan (positron emission tomography/computed tomography scan) can be benign or malignant and a tissue biopsy is needed for diagnose. A recent study found that it is possible to distinguish benign lesions from lung cancer lesions using CTCs as a marker [83].

Studies report that CTCs are present in 10% to 55% of early-stage (stage I to III) breast cancer patients and are associated with poorer outcomes [101,102]. CTC count can potentially be used for early diagnosis of cancer in patients that have an increased risk for cancer [85]. Patients with chronic obstructive pulmonary disease (COPD) have an increased risk of lung cancer [103]. It is possible to detect CTCs in COPD patients 1 to 4 years before the tumor was detectable with a CT-scan [85].

Blood contains CTCs from all tumor sites in the body and it has been suggested that this is a molecular proxy for the overall disease [104], in contrast to a tissue biopsy which only represents cancer at a particular site. This may open the possibility to use CTCs to assess the current tumor biology in order to monitor genetic aberrations that may influence treatment choices and personalize cancer treatment. CTCs are difficult to isolate compared to circulating tumor DNA (ctDNA) but can provide information on the genome as well as the transcriptome and proteome of cancer. Recent advances in whole genome amplification (WGA) have made it possible to interrogate a single CTCs with microarray-based comparative genomic hybridization (array-CGH) and high-throughput [105] and single-cell sequencing (SCS) [86,106,107].

Using amplicon-based sequencing it was found that aberrations in CTCs correlate with that found in the primary tumor, including mutations and amplifications in druggable genes [108]. CTC RNA sequencing data show that in breast cancer regulation patterns differ based on the location of the metastatic tumors [109]. Gulbahce, et al. [86] showed that genomic sequencing of CTCs can be used to analyze tumor heterogeneity and the detected somatic alterations provide information that may be used in stratify treatment. It is also possible to culture CTCs ex vivo, enabling drug sensitivity testing [110]. Clinical trials utilizing genomic aberrations in CTCs or expanded CTC drug sensitivity testing to stratify patient treatment are needed and may shed some light on its feasibility in the clinical setting.

The CTC studies done in Africa are listed in Table 1. Bahnassy, et al. [19] showed that the melanoma antigen-encoding gene 1 (MAGE1) and MAGE3 are expressed on the surface of CTCs from hepatocellular carcinoma (HCC) patients but not in healthy volunteers or chronic hepatitis C patients. Sayed et al. [18] evaluated the uses of CTC and cancer stem cells (CSCs) during the treatment of breast cancer. They found that CTC count at diagnosis can predict overall survival and CTC count after chemotherapy can predict disease-free survival and overall survival. High levels of CD44+/CD24− CSCs that remain after treatment was an indicator for recurrence [18]. Increased levels of CD133, a marker for stem cells and cancer stem cells, were associated with higher stage tumors and poor prognosis in HCC patients [20].

### 2.2. Circulating Tumor DNA

Mandel [111] first identified cell-free DNA (cfDNA) in 1948 and about 3 decades later Leon, et al. [112] found an increased level of cfDNA in the circulation of cancer patients. The increased level of cfDNA cannot only distinguish cancer patients from healthy individuals [113] but can also differentiate patients with malignant tumors from those with benign tumors (prognostic ability) [114,115].

In addition to being found in the blood, cfDNA is also found in urine, cerebrospinal fluid, saliva, and breast milk [116]. cfDNA is highly fragmented, it measures between 150 and 200 bp with an average length of 167 bp [117,118]. Apoptosis and/or necrosis are considered to be the main sources of cfDNA, although the full mechanisms by which cfDNA is released into circulation is not completely understood [118,119]. DNA fragments released from tumor cells are referred to as circulating tumor DNA (ctDNA). ctDNA has a longer fragment size than DNA fragment released from normal cells [120,121]. The fragment size of cfDNA is used to calculate the DNA integrity index. DNA integrity index is the ratio of long to short DNA fragments [122]. DNA integrity index is used along with cfDNA levels to diagnose cancer, monitor treatment response, and predict tumor stages [123].

DNA fragment size between different tumor types also differs largely due to metabolic and biological differences among tumors [124]. For example, the DNA fragment size from brain cancers reflects the filtration effect of the blood–brain barrier [124].

The half-life of cfDNA is approximately 2 h after which it is cleared from the circulation [125]. This means that cfDNA analysis can provide a real-time view of the genetic landscape that includes all the tumors in a patient (primary and metastatic). Studies have shown that DNA fragments released from tumors into circulation harbor tumor-specific aberrations, including mutations in tumor suppressors and oncogenes, microsatellite instability, DNA methylation, loss of heterozygosity and copy number variation [126,127,128,129,130]. Next-generation sequencing (NGS) can be used to identify all known and unknown genomic aberrations but is time-consuming and expensive. PCR-based techniques, such as real-time PCR or droplet PCR, is less expensive and faster but can only be used to assess known aberrations [131].

Wyatt, et al. [132] showed that driver DNA mutations found in metastatic tissue biopsies were concurrently present in the cfDNA. They concluded that for most patients, analyzing genetic alterations in cfDNA is sufficient to identify driver DNA alterations for managing metastatic castration-resistant prostate cancer [132]. Genetic mutations detected in CTC and ctDNA from the same patient show high concordance (<73%), although complimentary assessment may be beneficial to assess the dynamic tumor profile [133].

The FDA has approved two tests based on cfDNA for cancer management, the first is the Cobas EGFR Mutation Test v2 (Roche Molecular Diagnostics, Basel, Switzerland). The Cobas EGFR Mutation Test is a real-time PCR test that can detect and quantify 42 mutations on the epidermal growth factor receptor (EGFR) gene [134]. This test was approved to guide treatment decisions in non-small cell lung cancer (NSCLC). When compared to tissue-derived DNA results, the sensitivity of this cfDNA test is 72.1% and the specificity is 97.9% [135]. The second is the Epi proColon test, a real-time PCR test that detects hypermethylation in the promoter region of the Septin 9 gene (SEPT9) [136]. This test is the first approved blood-based screening test for colorectal cancer. The sensitivity of the test is between 71.1% and 95.6% and the specificity is between 81.5% and 99% [137].

Additionally, the AmoyDx Super-ARMS EGFR mutation test has been approved by the Chinese FDA for the detection of EGFR mutations in lung cancer. Four cfDNA-based tests have been approved for the EU market. They are the Qiagen Therascreen EGFR RGQ Plasma PCR kit (for detection of EGFR del19 and EGFRL858R in lung cancer); Sysmex Inostics OncoBEAM RAS CRC Kit (for detection of KRAS and NRAS mutations in colorectal cancer); Idylla™ ctKRAS Mutation Test (for detecting KRAS mutations in metastatic colorectal cancer patients), and the Idylla™ ctNRAS-BRAF mutation test (for detecting NRAS and BRAF mutations in metastatic colorectal cancer patients) [138]. A number of studies have investigated cfDNA in cancer management in Africa (Table 1). Ibrahim, et al. [30] found that both qualitative (fragment size) and quantitative aspects of cfDNA are associated with prognosis, metastasis, and treatment responses of Egyptian breast cancer patient. Fawzy, et al. [29] studied the role of cfDNA and DNA integrity in patients with metastatic prostate cancer and found cfDNA to be a potential non-invasive biomarker for screening and monitoring metastasis in prostate cancer patients. A recent study by Marchio, et al. [26] used droplet digital PCR technique to detect TP53 R249S mutants in cfDNA of HCC patients from the Central African Republic and Cameroon. The study suggested that the technique may be used for diagnosis and to conduct public health surveys on populations at risk of HCC [26].

### 2.3. Circulating Tumor RNAs

For decades, the concept of extracellular RNA has been one of the major focuses of scientific research. One of the most important studies was reported by Stroun and co-workers in 1978, who demonstrated that RNAs are released from cells into the culture medium [139]. Following this study, other studies have demonstrated that free-circulating RNAs can be released in the bloodstream of healthy people or cancer patients within particles-associated vesicles such as exosomes, microvesicles, and apoptotic bodies [123,140]. These vesicles protect the free-circulating RNAs from ribonucleases degradation and confer their stability [141]. Circulating RNAs do not result from random degradation but regulated cleavage and may play a specific role in cell physiology and also in cell-to-cell communication [142,143]. Circulating RNAs are detectable in human body fluids such as plasma, serum, and urine and have also been implicated in some disease outcomes like cancers [123]. Sensitive techniques like droplet digital PCR and RNA sequencing have also been recently developed and are used for circulating RNA detection [144]. These characteristics make circulating RNAs a potential biomarker in cancer management. Circulating RNAs include coding RNAs and non-coding RNAs (long non-coding RNAs and microRNAs) will be discussed in the sections below.

#### 2.3.1. Circulating Coding RNA

Circulating messenger RNAs (mRNAs) have been detected in body fluids of various cancer patients. These circulating mRNAs are associated with several cancer types such as breast, gastric, prostate, and colon cancers [145,146,147,148]. Many studies have shown the use of these circulating mRNAs as potential biomarkers in cancer patients for early detection and cancer progression monitoring [149,150].

In Africa, only a few studies have been reported. An Egyptian study investigated the role of transforming growth factor-beta 1 (TGF-β1) and Golgi protein 73 (GP73) circulating cell-free mRNAs as a potential biomarker for HCC [70]. The authors reported that TGF-β1 and GP73 mRNA expression was elevated in the serum of HCC patients compared to the control group. They also found that alpha-fetoprotein (AFP), which is usually measured for monitoring of patients with high HCC risk, showed a lower expression level than TGF-β1 and GP73. Abdelgawad, et al. [69] reported that the GPC3 level was elevated in the serum of all HCC patients compared to control subjects. The study also showed that measurement of the GPC3 level in serum of Egyptian patients with HCC is more sensitive than the currently used marker AFP.

#### 2.3.2. Long Non-Coding RNAs

Long non-coding RNAs (lncRNAs) represent a class of nucleic acid transcripts that are longer than 200 nucleotides in length and are not coding for any protein [151]. They have the potential to regulate various biological events such as cell differentiation, proliferation, migration, and invasion [152]. They are implicated in tumorigenesis with oncogene or tumor suppressor roles [152]. lncRNA profiles are more organ- and tumor-specific than other RNA entities and it was reported that circulating lncRNAs could reflect the pathological and physiological change of cancer patients [153,154]. A study by Xie, et al. [155] showed that lncRNAs SOX2OT and ANRIL were upregulated in lung cancer patients in comparison to healthy controls and therefore postulated that they could be good circulating markers for non-small cell lung cancer prognosis [155]. Lv, et al. [156] also demonstrated that high level of lncRNA HOTAIR in the serum of breast cancer patients induces less response to neoadjuvant chemotherapy, hence highlighting HOTAIR as a biomarker for the monitoring of breast cancer treatment.

The only non-coding RNA clinically approved test is the PROGENSA^®^ PCA3 urine test for prostate cancer screening in patients with one or more negative biopsies with a sensitivity and specificity of 48.4% and 78.6%, respectively [157]. This test detects the non-coding RNA prostate cancer antigen 3 (PCA3) in post-digital rectal exam first catch urine [158].

Only a few studies (Table 1) have investigated the role of lncRNAs as biomarkers for cancer diagnosis in Africa. Zidan, et al. [71] reported that MALAT1 (metastasis associated lung adenocarcinoma transcript 1) expression is increased in serum of Egyptian breast cancer patients. The study found MALAT1 to be more sensitive than CA15-3 which is the current marker for breast cancer [71]. Hashad, et al. [74] showed higher expression level of lncRNA H19 in the serum of gastric cancer patients when compared with the healthy control group and highlighted the possibility of using lncRNA H19 as a biomarker for gastric cancer diagnosis [74]. El-Tawdi, et al. [73] showed lncRNA-CTBP as a biomarker for HCC diagnosis. The study proposed the use of a panel of markers including lncRNA-CTBP, miR-16-2, miR-21-5p, and LAMP2 for best sensitivity and specificity of HCC diagnosis [73].

#### 2.3.3. Circulating microRNAs

MicroRNAs (miRNAs) are endogenous non-coding RNA transcripts with a length around 18 to 24 nucleotides [159] which play an important role in cell physiology by modulating gene expression at post-transcriptional level. They recognize their target messenger RNA by binding completely or incompletely to the 3′-untranslated region (3′-UTR) and induce their effect either via translational repression or mRNA degradation [160]. MicroRNAs are actively released from cells into various human body fluids like plasma, serum, urine, and saliva and their expression level is correlated with disease progression and physiological states [159,161,162].

Circulating microRNAs are packed within vesicles or associated with RNA binding proteins and are stable in bio-fluids [163]. In extracellular vesicles about half of the RNA are microRNAs [164]. Previous studies have proposed circulating miRNAs as non-invasive biomarkers for human diseases like cancer, mainly due to their high stability in bio-fluids and their expression level that correlated with the disease stage [165,166]. By comparing the profile of miRNAs in healthy subjects and cancer patients, many studies have observed alterations of miRNAs expression in many human tumors with the potential role of determining cancer outcome [167,168,169].

In the context of Africa, Motawi, et al. [170] reported that circulating miR-21 and miR-221 can be used to discriminate between breast cancer patients and healthy subjects in Egyptian women. The study showed that the expression of the two circulating miRNAs is higher in breast cancer cases compared to healthy persons. A study by Fattah, et al. [171] confirmed circulating miR-21 as a potential diagnostic marker for breast cancer in the Egyptian population. Circulating miRNA has also been used as a diagnostic marker for HCC. Alnoanmany, et al. [62] demonstrated circulating miR-21 was highly expressed in Egyptian HCC patients compared to the control group. They found that its use as a diagnostic tool for HCC is more sensitive than AFP, the current diagnostic marker for HCC [62]. Elhamamsy, et al. [55] in their study on Egyptian acute myeloid leukemia (AML) patients, showed that the expression level of circulating miR-92a, miR-143, and miR-342 were downregulated compared to control individuals. The study demonstrated that these circulating miRNAs have sensitivity and specificity high enough to be good markers for AML [55]. Several circulating miRNAs were identified as biomarkers for colorectal carcinoma (CRC). Zekri, et al. [61] compared the miRNA profile between CRC patients and healthy controls and found that circulating miR-17, miR-19a, miR-20a, and miR-223 were up-regulated in CRC patients. The circulating miRNAs found by Zekri, et al. [61] were shown to demonstrate high diagnostic performance which may be useful biomarkers for the screening of CRC and monitoring tumor dynamics.

### 2.4. Other Circulating Molecules

#### 2.4.1. Exosomes

The three main groups of extracellular vesicles released by cells are microvesicles, apoptotic bodies, and exosomes [172]. Exosomes are the best characterized and the most studied of these three types of extracellular vesicles [173]. Exosomes are small round vesicles with a size range of 40–150 nm in diameter [174,175]. Exosomes originate from endosomes and are actively produced and secreted by different types of cells including tumor cells [174,175]. Exosomes have been identified in blood, urine, cerebrospinal fluid, saliva, breast milk, pleural effusions, and nasal secretions [176,177,178].

Exosomes are employed by cells as vehicles to transmit molecular messages between homotypic and heterotypic cells and affect the phenotype of the recipient cells [175,179]. The exosome cargo includes a wide range of molecules such as proteins, lipids, mRNA, non-coding RNA, miRNA, and DNA. Plasma from cancer patients generally contains higher levels of exosomes compared to healthy control individuals, sustaining that tumor cells secrete more exosomes than normal cells [175]. Tumor-released exosomes exert their functions through cell–cell communications and impact malignant transformation, angiogenesis, immune response, and metastatic spread [180,181].

Accordingly, exosomes have a strong potential as blood or urine-based biomarkers for diagnostic prognostic and therapeutic management of cancer [175,179]. Recent studies highlight the relevance of tumor-secreted exosomes for cancer progression, response to therapy, and the possibility that different tumor types may secrete exosomes with unique cargos reflecting the tumor phenotype and clinical behavior. Furthermore, the exosome content in terms of DNA, RNA, and proteins gives insights into the intrinsic characteristic of tumors and can be exploited for personalized medicine approaches.

Despite their high potential, few studies have been done on the role of exosomes in the diagnosis and prognosis of cancer in Africa. An Egyptian study by Khalil, et al. [76] showed that exosomal lncRNA-RP11-510M2.10 can be used as a diagnostic and prognostic marker for lung cancer. Abd El Gwad, et al. [75] in their study showed that the accuracy of early diagnosis of HCC can be significantly improved using serum exosomal miR-1262, lncRNA-RP11-513I15.6, and AFP.

#### 2.4.2. Circulating Proteins and Peptides

Recent discoveries on new protein and peptide biomarkers have marked a new horizon in their use in non-invasive cancer diagnosis [182,183]. Several protein markers such as CA19-9, AFP, CEA, CA15-3, PSA, CA125, are in use clinically to boost the diagnosis and monitoring of cancer. Not all of these protein markers have a high specificity to cancer and the need to discover novel biomarkers that are highly specific and sensitive remains.

Numerous studies have evaluated the use of protein biomarkers in African populations; with some aiming at the discovery of novel protein biomarkers [184,185,186,187,188,189,190,191,192,193,194,195,196,197,198,199,200]. A study by Adeola, et al. [15] identified 73 urinary proteins as potential biomarkers for prostate cancer in the South African population. Abdel Wahab et al. [184] identified 33 deregulated proteins in Egyptian cohort that could be used as a prognostic signature for hepatitis C-virus related HCC. A recent review by Adeola, et al. [201] highlights the prospects of using proteomic biomarkers in Africa.

## 3. Challenges to Implementation of Liquid Biopsy Technology in Africa

Even though liquid biopsy techniques have been used for the diagnosis of various cancer types in the advanced western world [201], its routine clinical use has not received wide coverage in African healthcare settings. This can be due to limiting factors such as lack of skilled personnel, poor health infrastructure, and poor government policies [201]. As a relatively new technology, a potential drawback of liquid biopsy in Africa is that it’s market products would not be affordable to the majority of low-income countries; and it may not be easily available for the general population. A typical example is Kenya which recently became the third African country after South Africa and Tunisia in which the use of liquid biopsy is commercially made available to the public [202]. The estimated cost for each test was considered to be seventy thousand Kenyan Shillings (about $7000) which is practically unaffordable to middle- and low-income earners that form the majority of the country’s population [202].

In addition, it may be difficult to ascertain the sensitivity, specificity, and efficacy of liquid biopsy methods in African patients, as most of the available products have not been tested in African populations. Also, most of the technology used for detecting genetic mutations and alterations are developed using non-African populations. This is particularly concerning because of the wide geographic/latitudinal variations in pathogenesis and natural history of cancers in Africa [203]. Therefore, ad hoc designed clinical studies in African populations are needed to address these questions and give insight on population-specific biomarkers for early diagnosis and monitoring.

Generally, parallel detection of various liquid biopsy analytes may be challenging from a small volume of blood [204]; and this integrated liquid biopsy investigation has to be significantly improved to provide in-depth information on tumor genotypes/phenotypes. The issue of low-frequency mutant allele in the analysis of cell-free nucleic acid is also a huge challenge due to variability among early-stage tumors [205]. Thus, adequate, cutting-edge, basic, translational, and clinical research in the African continent should be supported in this important and emerging field, which has enormous potential and social impact for improving public health at sustainable costs. For this technology to emerge in Africa, great capacity needs to be built in terms of specialized personnel training and infrastructure building, not only in the domain of molecular biology and clinical research but also in computational biology and bioinformatics, to standardize and validate potential biomarkers [204,206].

## 4. The Prospect for Liquid Biopsy in Africa

In spite of the challenges, liquid biopsy has emerged as a rapid, reliable, and minimally invasive cancer screening solution, with high specificity and sensitivity for cancer diagnosis and monitoring [205,206,207,208]. In western world settings, the use of liquid biopsy options like CTCs and ctDNA has been a cutting-edge technology that has improved the detection and monitoring of cancer in a small amount of blood samples [209,210,211,212,213]. It proved that real-time monitoring of disease progression can be used to personalize treatment, which can increase effectiveness and reduce the cost of treatment. Indeed, new technologies for detection of gene mutations, genetic rearrangements, and additional cancer-specific biomarkers applied to liquid biopsy promise further cost-cutting.

Cancer in Africa is a public health menace. Most cancer cases are diagnosed late in Africa [214]. For example, in Zimbabwe 80% of cervical cancer patients presented with advanced disease [215] and in Tanzania, more than 70% of breast cancer patient were diagnosed at stage III and above [216]. Factors such as limited knowledge of signs and symptoms of cancer, limited screening facilities, and fear of surgery are some of the major barriers to early presentation and cancer diagnosis in Africa [217,218,219]. Liquid biopsy could help in addressing these challenges as it is non-invasive and does not require surgical facilities not always available in African settings.

As shown in the developed world, liquid biopsy can be an implementable and promising concept that would non-invasively boost the prompt identification of cancers and reduce the morbidities and mortalities associated with the late/end-stage diagnosis of cancer. The initial burden and high costs due of the introduction of this high-tech, cutting-edge technology should be sustained by national and international collaborative research and health care programs aimed at reducing disparities both among and within African countries. Eventually, health care costs in African countries would be reduced by implementing policies and technologies leading to easier access to diagnostic procedures, early detection, and effective clinical predictive measures. Additionally, liquid biopsy approaches would promote the practice of evidence-based precision medicine in Africa, applying therapies when needed, and avoiding the human and societal costs of under- and over-treatment. Ultimately, it is imperative that more resources are channeled towards African-based studies on the molecular basis of cancer, enabling technologies and personnel training with the goal of developing genomic, proteomic, and cell-based biomarkers that are specific to the African population. This will make easy and early diagnosis a reality and help to improve the management of cancer in the African continent.

## Figures and Tables

**Table 1:** African-based studies on the role of circulating molecules in cancer diagnosis.

Samples	Study Design	Cancer Type	Downstream Analysis	Country	References
**CTC**					
**Blood**	75 BC patients 20 healthy controls	Breast cancer	Circulating endothelial progenitor cells count CD14, CD133 and VEGFR2 expression levels (flow cytometry)	Egypt	Montaser, et al. [16]
**Blood**	50 BC patients 14 healthy controls	Breast cancer	mRNA expression levels (qPCR)	Egypt	Elnagdy, et al. [17]
**Blood**	51 BC patients	Breast cancer	CTC and CSC count (flow cytometry)	Egypt	Sayed, et al. [18]
**Peripheral blood**	70 HCC patients 30 CHC patients 33 healthy controls	Liver cancer	CTC and CSC countProtein expression levels of CK19, CD133, CD90 (flow cytometry)	Egypt	Bahnassy, et al. [19]
**Blood**	50 HCC patients 20 healthy controls	Liver cancer	CTC count (flow cytometry)	Egypt	Mansour, et al. [20]
**Blood**	40 BC patients	Breast cancer	CTC and CSC count (flow cytometry)	Egypt	Zedan, et al. [21]
**Blood**	50 BC patients 30 healthy controls	Breast cancer	mRNA expression levels (qPCR)	Egypt	Ebeed, et al. [22]
**Blood**	36 CRC patients 18 healthy controls	Colorectal cancer	mRNA expression levels (qPCR)	Egypt	Teama and Agwa [23]
**Blood**	147 BC patients 94 healthy controls (41 U.S. healthy volunteers)	Breast cancer	mRNA expression levels (qPCR)	Senegal	Zehentner, et al. [24]
**Peripheral blood**	143 primary melanoma patients	Melanoma	The use of qPCR to determine the presence of tyrosinase mRNA in peripheral blood	South Africa	Hanekom, et al. [25]
**cfDNA**					
**Plasma**	195 HCC patients 263 CLD control patients 49 healthy controls	Liver cancer	cfDNA mutational analysis using droplet digital PCR	Cameroon, Central African Republic	Marchio, et al. [26]
**Plasma**	40 BC patients 10 healthy controls	Breast cancer	cfDNA quantification and Integrity index using qPCR	Egypt	Hussein, et al. [27]
**Serum**	60 LC patients 40 COPD patients 40 healthy controls	Lung cancer	cfDNA quantification and Integrity index using qPCR	Egypt	Soliman, et al. [28]
**Plasma**	50 PCa patients 25 BPH patients 30 healthy controls	Prostate cancer	cfDNA quantification and Integrity index using qPCR	Egypt	Fawzy, et al. [29]
**Serum**	40 BC patients 40 healthy controls	Breast cancer	cfDNA quantification using qPCR	Egypt	Ibrahim, et al. [30]
**Serum**	50 CRC patients 10 colonic polyps’ patients 20 healthy controls	Colorectal cancer	cfDNA quantification and Integrity index using qPCR	Egypt	El-Gayar, et al. [31]
**Plasma**	50 BC patients 30 benign breast lesions 20 healthy controls	Breast cancer	Quantification of cfDNA andmtDNA using multiplex qPCR	Egypt	Mahmoud, et al. [32]
**Plasma**	120 cancer patients 120 patients with benign diseases 120 healthy controls	Breast, Lung, Colon and Liver cancers	cfDNA quantification and Integrity index using qPCR	Egypt	Zaher, et al. [33]
**Plasma**	42 BC patients 30 benign lesion patients 27 healthy controls	Breast cancer	cfDNA quantification and Integrity index using qPCR	Egypt	Hashad, et al. [34]
**Serum**	25 HCV-related HCC patients 25 chronic HCV patients 15 healthy controls	Liver cancer	cfDNA quantification and Integrity index using qPCR	Egypt	El-Shazly, et al. [35]
**Plasma**	28 HCC patients	Liver cancer	Methylation profile determined for five genes using qPCR	Egypt	Iyer, et al. [36]
**Serum**	20 NHL patients 20 healthy controls	non-Hodgkin’s lymphoma	cfDNA quantification using Fluorometric assay	Egypt	Hosny, et al. [37]
**Serum**	76 HCC patients 110 CLD patients 69 healthy controls	Liver cancer	cfDNA quantification and sequencing of the positive RFLP fragments using nested PCR	Egypt	Hosny, et al. [38]
**Plasma**	216 HCC patients 121 liver cirrhosis patients 408 healthy controls	Liver cancer	cfDNA quantification and sequencing using nested PCR	Gambia	Kirk, et al. [39]
**Plasma**	29 HCC patients	Liver cancer	cfDNA quantification and sequencing using nested PCR	Gambia	Szymanska, et al. [40]
**Plasma**	12 PCa patients 10 healthy controls	Prostate cancer	cfDNA quantification and parallel tagged sequencing	South Africa	van der Vaart, et al. [41]
**Plasma**	1 BC patient 1 healthy control	Breast cancer	Cloning and sequencing of cfDNA	South Africa	van der Vaart and Pretorius [42]
**miRNA**					
**Serum**	65 LC patients 29 pulmonary tuberculosis patients 29 pneumonia 37 healthy controls	Lung cancer	Expression levels of miR-21, miR-155, miR-182, and miR-197 assessed using qPCR	Egypt	Abd-El-Fattah, et al. [43]
**Serum**	60 HCV-related HCC patients 60 HCV-related liver cirrhosis patients 60 healthy controls	Liver cancer	Expression levels of miRNAs determined using qPCR	Egypt	Ali, et al. [44]
**Serum**	60 HCC patients 30 healthy controls	Liver cancer	Expression levels of microRNAs 191, 203 and 335 determined using qPCR	Egypt	Ezzat, et al. [45]
**Plasma**	45 LC patients 40 healthy controls	Lung cancer	The expression level of miR-21 and miR-23a was detected by qPCR	Egypt	Hetta, et al. [46]
**Serum**	60 ovarian cancer patients 30 healthy controls	Ovarian cancer	Serum miR-21 levels were measured by TaqMan-qPCR	Egypt	Mahmoud, et al. [47]
**Serum**	35 CRC patients 51 patients with benign lesions 101 healthy controls	Colorectal cancer	The expression of miR-210, miR-21 and miR-126 was performed using qPCR	Egypt	Sabry, et al. [48]
**Serum**	106 BC patients 49 benign breast lesion patients 40 healthy controls	Breast Cancer	The expression level of miR-335 was detected by qPCR	Egypt	Swellam, et al. [49]
**Serum**	137 BC patients 60 benign breast lesion patients 38 healthy controls	Breast cancer	miRNAs expression levels were determined using reaction qPCR	Egypt	Swellam, et al. [50]
**Serum**	30 HCC patients 20 healthy controls	Liver cancer	lncRNA GAS5 and miR-34a expression level measured using qPCR	Egypt	Toraih, et al. [51]
**Blood**	9 CHC patients 6 liver cirrhosis patients 9 HCC patients 8 healthy controls	Liver cancer	miRNAs expression levels were determined using reaction qPCR	Egypt	Zekri, et al. [52]
**Plasma**	60 HCC patients 60 CHC patients 60 healthy controls	Liver cancer	miRNA expression levels assessed using qPCR	Egypt	Demerdash, et al. [53]
**Serum**	224 HCC patients 250 CHC patients 84 healthy controls	Liver cancer	miRNAs (hsa-miR-1269, hsa-miR-125b, hsa-miR-138, hsa-miR-214-5p, hsa-miR-494, hsa-miR-375 and hsa-miR-145) were assessed using qPCR	Egypt	Elemeery, et al. [54]
**Plasma**	65 AML patients 50 healthy controls	Acute myeloid leukemia	Expression of miR-92a, miR-143 and miR-342 was measured using qPCR	Egypt	Elhamamsy, et al. [55]
**Serum**	64 CRC patients 27 healthy controls	Colorectal cancer	Expression levels of miR-92a, miR-375, and miR-760 assessed using qPCR	Egypt	Elshafei, et al. [56]
**Serum**	23 HCC patients 25 post-HCV liver cirrhosis patients 30 HCV patients 10 healthy controls	Liver cancer	miRNA expression levels using qPCR	Egypt	Khairy, et al. [57]
**Plasma**	70 bladder cancer patients 62 healthy controls	Bladder cancer	Expression levels of miR-92a, miR-100 and miR-143 measured using qPCR	Egypt	Motawi, et al. [58]
**Serum**	60 HCC patients 40 CHC patients 30 healthy controls	Liver cancer	Expression levels of miRNA-122 and miRNA-222 assessed using qPCR	Egypt	Motawi, et al. [59]
**Peripheral blood mononuclear cells**	85 ALL patients 25 healthy controls	Acute lymphoblastic leukemia	Expression levels of miR-92a, miR-100 and miR-143 were measured using qPCR	Egypt	Swellam and El-Khazragy [60]
**Serum**	30 CRC patients 18 IBD patients 18 colonic polyps’ patients 24 colonic symptoms patients 100 CRC patients (validation)	Colorectal cancer	miRNAs expression levels were determined using reaction qPCR	Egypt	Zekri, et al. [61]
**Blood**	30 HCC patients 20 HCV patients 20 healthy controls	Liver cancer	miRNA expression levels assessed using qPCR	Egypt	Alnoanmany, et al. [62]
**Urine**	188 Bladder cancer patients 88 Benign bladder lesions 92 healthy controls	Bladder cancer	miR-210, miR-10b, miR-29c, miR-221, and miR-23a expression levels assessed using qPCR	Egypt	Eissa, et al. [63]
**Serum**	40 HCC patients 40 HCV patients 20 Healthy controls	Liver cancer	miRNA expression levels using qPCR	Egypt	El-Abd, et al. [64]
**Serum**	120 BC patients 50 healthy controls	Breast cancer	Expression levels of miRNAs (miR10b, miR34a, miR155, miR195 and miR16) determined using qPCR	Egypt	Hagrass, et al. [65]
**Serum**	112 HCV-related HCC patients 125 HCV-related CLD patients 42 healthy controls	Liver cancer	Expression miRNA was measured using qPCR	Egypt	Motawi, et al. [66]
**Urine**	32 HCC patients with post-HCV infection 74 chronic HCV patients 12 healthy controls	Liver cancer	miRNA whole-genome expression profiling and relative expression profiling for candidate miRNAs using qPCR	Egypt	Abdalla and Haj-Ahmad [67]
**Serum**	20 Inflammatory BC patients 20 non-inflammatory BC patients 20 healthy controls	Breast cancer	TaqMan qPCR was performed to detect the circulating expression of miRNAs	Tunisia	Hamdi, et al. [68]
**mRNA**					
**Serum**	40 HCC patients 10 healthy controls	Liver cancer	mRNA expression levels using qPCR	Egypt	Abdelgawad, et al. [69]
**Serum**	25 HCC patients 15 healthy controls	Liver cancer	mRNA expression levels using qPCR	Egypt	Ibrahim, et al. [70]
**lncRNAs**					
**Serum**	80 BC patients 80 healthy controls	Breast cancer	mRNA expression levels using qPCR	Egypt	Zidan, et al. [71]
**Serum**	120 CRC patients 30 adenomatous polyps’ patients 96 healthy controls	Colorectal cancer	Serum expression levels of lncRNAs and miRNA using qPCR	Egypt	Shaker, et al. [72]
**Serum**	78 HCC patients 36 CHC patients 44 healthy controls	Liver cancer	mRNA expression levels using qPCR	Egypt	El-Tawdi, et al. [73]
**Plasma**	32 gastric cancer patients 30 healthy controls	Gastric cancer	mRNA expression levels using qPCR	Egypt	Hashad, et al. [74]
**Exosomes**					
**Serum**	60 HCC patients 42 CHC patients 18 healthy controls	Liver cancer	Expression of exosomal RNA using qPCR	Egypt	Abd El Gwad, et al. [75]
**Serum**	20 LC patients	Lung cancer	Expression of exosomal RNA using qPCR	Egypt	Khalil, et al. [76]

Abbreviations: ALL—Acute lymphoblastic leukemia, AML—Acute myeloid leukemia, BC—Breast cancer, BPH—Benign prostatic hyperplasia, CHC—Chronic hepatitis C, CLD—Chronic liver disease, COPD—Chronic obstructive pulmonary disease, CRC—Colorectal cancer, CSC—Cancer stem cell, HCC—Hepatocellular carcinoma, HCV—Hepatitis C-Virus, IBD—Inflammatory bowel disease, LC—Lung cancer, mtDNA—mitochondrial DNA, NHL—Non-Hodgkin’s lymphoma, PCa—Prostate cancer, PC—Pancreatic cancer, qPCR—Quantitative real-time PCR.

**Table 2:** Comparison of the circulating biomarkers, CTC, cfDNA, circulating tumor RNA and Exosomes in cancer management.

Analysis Capability	CTC	cfDNA	Circulating Tumor RNA	Exosomes
Genomic mutations	Yes	Yes	Yes	Yes
RNA profiling	Yes	No	Yes	Yes
Phenotypic studies of tumor cell	Yes	No	No	No
Proteomic analysis	Yes	No	No	Yes
**Clinical Applications**				
Clinical trials	Phase IV	Phase IV	Phase IV	Phase II
Clinical approved techniques	CellSearch	Cobas^®^ EGFR Mutation Test v2 assay Epi proColon test AmoyDx Super-ARMS EGFR mutation test Therascreen EGFR RGQ Plasma PCR kit Therascreen PIK3CA RGQ PCR Kit Sysmex Inostics OncoBEAM RAS CRC Kit Idylla™ ctKRAS Mutation Test Idylla™ ctNRAS-BRAF mutation test	Progensa™ PCA3	No
Cost of clinical use (outside Africa)	$350	$170–470	$220	-
